# Maternal Obesity and the Fetal Origins of the Metabolic Syndrome

**DOI:** 10.1007/s12170-012-0257-x

**Published:** 2012-08-14

**Authors:** Jwan Rkhzay-Jaf, Jacqueline F. O’Dowd, Claire J. Stocker

**Affiliations:** Clore Laboratory, University of Buckingham, Hunter Street, Buckingham, MK18 1EG UK

**Keywords:** Maternal overnutrition, Developmental programming, Type 2 diabetes, Cardiovascular disease, Obesity

## Abstract

Over recent decades there has been a rapid rise in metabolic disorders throughout the world. Whilst lifestyle and societal habits have contributed to the obesity epidemic, there is now increasing evidence that the early developmental environment of an infant can play a pivotal role in the ‘programming’ of an adverse physiological phenotype in later life. Clinical evidence highlights that maternal over-nutrition and/or obesity during pregnancy presents not only adverse effects on maternal health, but also persistent and deleterious effects in the developing child. Animal models are providing essential information into the underlying cellular and molecular mechanisms that contribute to this adverse phenotype. The use of this information will aid our understanding of the programming signals related to maternal and paternal over-nutrition and the improved healthcare for both mother and infant.

## Introduction

Overweight and obesity are considered to be the fifth leading risks for global deaths with at least 2.8 million adults dying each year as a result. In 2008, 1.5 billion adults aged 20 years and older were overweight; of these over 200 million men and nearly 300 million women were obese. In addition, 44 % of the diabetes, 23 % of the ischemic heart disease. and between 7 % and 41 % of certain cancer burdens are attributable to overweight and obesity. Its growing prevalence is therefore one of the major health care issues of this century [1]. In 2010, 43 million children under 5 were estimated to be overweight and childhood obesity tracks strongly to adolescent and adulthood obesity [2]. Childhood overweight and obesity, once considered a problem of high-income countries, are now rising in low and middle income countries, particularly in urban areas with close to 35 million overweight children living in developing countries and 8 million in developed countries. Of particular concern is the growing prevalence of obesity in women of child-bearing age, as not only does this have health implications for them, but also there is increasing evidence that obesity during pregnancy and lactation can have long term effects on the health of the child [3, 4].

Genome-wide association studies have given insight into the importance of an individual’s genetic makeup and their predisposition to metabolic disease [5]. However, the dramatic rise in obesity indicates that this must be driven by environmental factors that affect dietary and physical activity patterns. In addition, it is now well recognized that the phenotype of an individual can be determined by its early developmental environment and in particular by the nutritional status of the mother. Such studies have led to the proposals of ‘developmental programming’ that describe how the conditions presented during a critical window of development can lead to the permanent programmed alterations in a physiological process.

## Early Life Programming

Epidemiological data worldwide has revealed a relationship between birth weight and the risk of developing cardiovascular and metabolic disease. Some of the earliest studies focused on the relationship between maternal under-nutrition and an infant being small for gestational age. Forsdahl proposed that poverty in early childhood could program the infant for low energy intake. If such an individual then consumed modern day, high energy-dense foods, this could be one reason why in recent times there is an increased incidence of cardiovascular disease [6]. Men exposed *in utero* to the Dutch Hunger Winter famine were more obese at age 19 if they were exposed to the famine during the first half of their mother’s pregnancy. In contrast, those who were exposed to the famine during the last trimester of pregnancy and in early postnatal life had reduced obesity [7]. Studies in Hertfordshire in the UK showed that men who were the smallest at birth were at increased risk of heart disease [8] and were more likely to be glucose intolerant or type 2 diabetic [9, 10]. These studies formed the basis of the ‘Thrifty phenotype hypothesis’ [11] that proposed that poor fetal nutrition leads to metabolic adaptations to maximize the chances of survival in conditions of on-going nutritional deprivation. Such adaptations would be beneficial if exposed to poor conditions after birth, but would not be suited if the postnatal environment provided plentiful nutrition, resulting in an increased risk of developing features of the metabolic syndrome. Other hypotheses have since been proposed to understand the observation that fetal nutrition leads to the permanent programming of metabolism and a maladapted adult phenotype if the fetus is born into conditions where the early and adult environments are ‘mismatched’. Of more relevance, however, to modern society in which maternal nutritional excess is more common, is the ‘developmental over-nutrition hypothesis’.

## Clinical Impact of Maternal Diet and Maternal Obesity and Postnatal Overnutrition

The prevalence of obesity is highest among children of obese parents [12]. Children of obese mothers are more at risk of overweight or obesity than those of obese fathers [13–15]. Maternal pre-pregnancy overweight has also been found to be as an independent risk factor for infant overweight and abdominal obesity [16]. Moreover, there are associations between pregnant maternal body mass index (BMI) and offspring BMI, adiposity, and insulin resistance [17–19], and also between maternal weight gain during pregnancy and offspring adiposity [20•]. Obesity in pregnancy is strongly associated with the development of gestational diabetes and there are many reports of associations between maternal diabetes and offspring diabetes [21, 22], and offspring obesity [23]. Recent clinical guidelines have reported that almost 1 in 5 UK pregnant women are now obese, which is a serious concern not only because of adverse pregnancy outcome, particularly gestational diabetes and fetal macrosomia, but also because of the reported associations with childhood risk of obesity, and insulin resistance. But possibly of greater concern are reports that moderate weight gain between successive pregnancies could result in an increase in birth weight [24]. Better-nourished mothers give birth to better nourished mothers-to-be, resulting in transgenerational obesity [25]. The interpretation is that susceptibility to obesity, type 2 diabetes, and cardiovascular disease is partly programmed by the environment during early pre and post natal life.

The early postnatal environment is a key time point for developmental programming to occur. Since physiological systems continue to develop and mature after birth, over-nutrition in this period will have considerable impact. Rapid postnatal growth following maternal malnutrition leads to obesity in later life and carries the highest risk of insulin resistance. Some reports suggest that rapid growth in the first few weeks of postnatal life is particularly disadvantageous [26], while other reports indicate that rapid growth of low birth weight infants in later childhood also increases their risk of obesity [27]. It is now known that rapid growth in itself is associated with an increased risk of elevated blood pressure, cardiovascular disease and type 2 diabetes, as well as a disproportionately high rate of fat deposition [28]. During this period dietary composition can affect growth patterns of the infant. Nutrient-enriched formula feeding is often used as an alternative or supplemental to breast feeding and can accelerate infant growth [26, 29]. Formula feeding increases the risk of obesity in childhood [26] as well as high cholesterol in adulthood [30] compared with breast feeding. Bottle-fed infants have higher total energy and protein intakes than breast-fed infants. So one possible explanation is that the plane of nutrition during the suckling period is a strong determinant for subsequent appetite regulation. Breastfed infants appear to have a greater ability to regulate food intake both when breastfeeding [31] and when eating solid food. Furthermore, breastfed infants show a reduced growth rate compared with formula-fed infants, are leaner, and are less susceptible to developing obesity, cardiovascular risk factors and hypertension [32].

The identification of the factors in the maternal environment that mediate the effects of maternal obesity and diet is of key importance to the development of clinical intervention strategies. Maternal hyperglycemia during pregnancy was thought to be one of the most important predictive factors of infant obesity and metabolic disease [33]. Now it is recognized that other maternal parameters associated with obesity and/or over-nutrition during pregnancy are involved including hyperinsulinaemia, hypertriglyceridaemia, and hyperleptinaemia. Maternal hyperglycaemia stimulates fetal insulin synthesis and increases fetal adiposity, which may permanently influence fetal adipocyte mass [34]. Maternal triglycerides, which are elevated in obese and insulin resistant women, will not cross the placenta but are hydrolysed by placental lipases [35] and may affect fetal fuel supply. Moreover, several reports have highlighted correlations between maternal insulin resistance, maternal plasma triglycerides, and offspring adiposity [14, 36]. Other studies in pregnant women and non-human primates have implicated the inflammatory state associated with maternal obesity as causing persistent influences on offspring metabolic function [37, 38]. Neonatal hyperinsulinaemia has been associated with increased adiposity in the infant [39].

## Animal Models of Developmental Programming

Whilst epidemiological studies are essential for establishing correlations between maternal nutritional status and offspring health, they are often complicated by genetic or environmental variables that confound correlation analysis. Animal models have therefore provided an invaluable insight into the underlying mechanisms of developmental programming because they allow intervention studies and also because genetic background can be strictly controlled. Such studies show strong parallels with the human observational studies, supporting a causal relationship between maternal obesity and offspring adiposity, glucose tolerance, insulin sensitivity, and cardiovascular disease. The majority of research has focused on sheep and rodents but rodents present a better model as they develop features of metabolic syndrome within months and are therefore more feasible for study. Non-human primates offer advantages because of the similarities of their developmental patterns to humans; however long gestation periods and high costs make them less available. When interpreting these studies, it is important to recognize that dietary manipulation in itself could introduce complications, as increasing one component could require the reduction of another. Offspring of high fat-fed dams have increased adiposity, insulin resistance, and hypertension [40], but here increasing the fat content reduces the carbohydrate content [41, 42], or protein content [43]. Modern day western diets are high in sugar as well as in fat and these obesogenic diets are available commercially. The offspring from these dams are hyperphagic, insulin resistant, glucose intolerant and hypertensive [41, 42]. The maternal ‘junk food’ diet also produces offspring with increased adiposity and hyperphagia for junk food after weaning [44]. All these diets have provided good evidence that maternal nutritional excess can contribute to this relationship [41–46].

Animal models have been used to specifically investigate the programming role of fuels and hormones that can pass directly from mother to fetus. Models of maternal diabetes have focussed on its effects on the pancreas leading to neonatal hyperinsulinemia and on the developing hypothalamus, leading to increased food intake and offspring obesity [47]. In the mouse, insulin and leptin were raised both on day 18 of gestation and at the end of lactation when fed an obesogenic diet [41]. This was associated with early elevated leptin and resistance to the action of leptin to reduce food intake in the offspring [48]. In the sheep increased placental fatty transporters has been associated with higher fetal triglyceride levels [49], enhanced cytokine expression in the placenta [50], and upregulated lipogenic genes in the adipose [51]. Similarly in mice, a high fat *in-utero* environment can increase the fetal triglyceride profile [52]. As in large for gestational age human fetuses, changes in placental morphology in overfed non-human primates have been observed [53], associated with increased nutrient delivery to the fetus for example of free fatty acids [54].

Rodent models have been used to investigate the effects of postnatal over-nutrition. The majority of studies have manipulated litter size post birth to alter the nutritional exposure. Reducing litter size results in hyperphagia, hyperinsulinemia, and cardiovascular risk in the offspring [55]; others report that rearing diet-induced obese rats in large litters protects against obesity [56]. Cross fostering has also been used to increase an infant’s nutritional plane in the suckling period and program susceptibility to obesity in adult life [57]. In these models pups suckled in large litters or whose dams are under-nourished are resistant to the development of obesity even when the pups are given a high fat diet from weaning [58]. In models of gestational diabetes, hyperglycemia during suckling results in obesity and insulin resistance in the infant in later life [59].

**Fig. 1:**
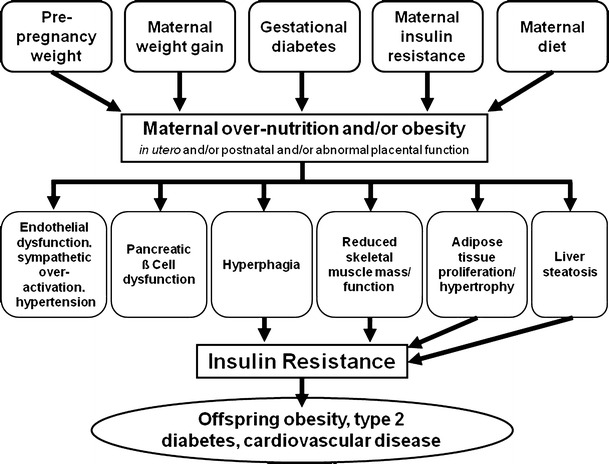
Common mechanistic pathways of developmental programming as a result of maternal over nutrition

Despite the wealth of data from a variety of models, a common offspring phenotype of increased adiposity, hyperphagia, insulin resistance, and hypertension is emerging (Fig. 1). The programming of obesity is a multifactorial process but similar mechanistic pathways are being revealed.

## Maternal Diet vs Maternal Obesity

In human studies it is difficult to dissect out the effects of maternal obesity from those of an obesogenic diet, but this can be addressed in animal models. A high fat diet throughout pre-gestation, gestational period, or postnatally will have different effects depending on the time the diet is introduced. The importance of maternal obesity independent of a difference in diet is supported by the observation that chow-fed wild-type offspring of the leptin receptor deficient (*db/db*) dam are obese and hyperinsulinaemic [60]. In contrast, there is evidence for the effects of maternal diet independent of increases in maternal weight. In studies where rat dams were fed a cafeteria-style or high fat diet initiated after conception so that during pregnancy and lactation the dams were not obese [43, 61], their offspring developed increased adiposity, hyperglycaemia, and hyperinsulinaemia. Most models however have studied the offspring from dams fed an obesogenic or high fat diet throughout pregnancy and lactation, leading to increased adiposity of offspring at weaning and glucose intolerance [41, 42]. It is now emerging that the postnatal diet can modify the programming of maternal obesity in utero. When offspring were exposed to maternal obesity during gestation and lactation and then maintained on a high fat diet after weaning, they rapidly gained weight and adipose mass [62]. Similarly female pups fed a formula diet immediately after birth, were obese, and obesity was then transmitted to the next generation even if these mice were maintained on a chow diet [63]. Maternal obesity and diet have been reported to have different effects on fetal growth. By varying the exposure of the dam either before gestation, resulting in maternal obesity or during gestation, it was reported that the obese in utero environment resulted in reduced fetal weight, whilst the obese gestational diet caused abnormal placental growth [64].

## Developmental Programming Mechanisms

The animal models of nutritional programming demonstrate common mechanisms of adult disease susceptibility with man, including alterations in appetitive behavior, accelerated loss of glucose tolerance and insulin sensitivity, changes in thermogenic capacity, and altered percentage fat mass and distribution.

### Central Mechanisms

The hypothalamus plays an essential role in the control of energy balance; its nuclei continue to differentiate postnatally [65] and hypothalamic neuropeptides can be permanently altered by the maternal and fetal dietary environment. Offspring of dams fed a fat rich diet are hyperphagic soon after weaning and show exaggerated feeding responses to orexigenic peptides and blunted signalling to satiety peptides. This could be one explanation for the observed changes in appetitive behavior [66] and food preferences [45] in the offspring [49]. Both leptin and insulin regulate long-term feeding behavior. Leptin acts on the arcuate nucleus in the hypothalamus to reduce hunger and increase energy expenditure and following administration to postnatal mice, leptin has been shown to deactivate orexigenic neuropeptide Y (NPY) and activate anorectic pro-opiomalanocortin neurones [67]. Rat pups from small litters show increased NPY expression and a reduced responsiveness to leptin [68, 69]. Leptin also plays a role in the neonatal development of the hypothalamus. Neuronal connection pathways in the arcuate nucleus are permanently disrupted in the leptin deficient obese mouse. Leptin treatment in adulthood is unable to reverse this neuro-anatomical defect [70] however treatment during the perinatal period completely restores the density and length of these neuronal projections [71]. During the early postnatal period there is a surge in circulating plasma leptin, independent of fat mass, which may serve as a key developmental signal to the hypothalamus to influence subsequent food intake and body weight [72]. Therefore any changes in leptin levels or leptin action [73] during this critical period may cause permanent alterations in the neuronal circuitry [74]. The maternal environment can influence the precise timing and hence the effect of this surge. Offspring of rat dams fed an obesogenic diet have an amplified and prolonged neonatal leptin surge, with attenuated leptin signalling before the onset of hyperphagia [48]. Similarly in sheep, whose lambs are born at an equivalent level of maturity to humans, a premature leptin peak is observed accompanied by increased appetite [75]. Therefore there may be a beneficial role for leptin in the reversal of the metabolic programming of maternal obesity, as it does in offspring of undernourished and normal dams where the leptin surge is reduced, both during gestation and lactation [76] or during suckling alone [77].

A neurotrophic role for insulin in hypothalamic development is now emerging. Suboptimal or neonatal hyperinsulinemia may result in the malformation of hypothalamic structures and their role in the control of food intake. The hyperphagia seen in models of maternal obesity may also be the consequence of dysregulated reward pathways. Food preferences and the motivation to eat highly palatable foods have been associated with the altered expression of the mesolimbic dopamine and opioid reward pathways [78].

### Peripheral Mechanisms

Programmed alterations in peripheral tissues such as muscle, adipose, and liver affect energy balance and glucose homeostasis. A progressive impairment of glucose homeostasis is highlighted in many animal models of maternal obesity. Many of the changes are due to changes in insulin sensitivity of the peripheral tissues resulting from a defect in insulin action downstream of the receptor [79]. In models of maternal obesity insulin resistance likely precedes beta cell failure [63]. The skeletal muscle is a major insulin sensitive tissue and abnormal development and function has been reported in a number of models [41]. Offspring of over-nourished dams exhibit reduced muscle cell proliferation and intramuscular lipid accumulation [44]. The maternal diet influences adipogenesis and the programming of adipocyte morphology and metabolism, with changes in the pattern of expression of key regulatory and functional genes, which are important determinants of fat distribution and accumulation. Persistent alterations in the expression of proteins involved in adipocyte development and lipolysis could result in permanent influences on adipocyte proliferation and hypertrophy [80]. An increased fat mass may be a compensatory mechanism to ensure that excess lipids are stored in adipocytes rather than ectopically; however an increase in adiposity results in increased insulin resistance and inflammatory responses. The liver plays a pivotal role in metabolism, elevated triglyceride levels and fatty liver have been associated with maternal over-nutrition [41] as well as the upregulation of genes involved in hepatic lipid biosynthesis [62, 81].

### Cardiovascular System

Studies of pregnant women and their infants found that those who exceeded the recommended weight gain during pregnancy give birth to children, who at age 9 presented with increased systolic blood pressure, C-reactive protein, lower high-density lipoprotein cholesterol, and increased BMI [82]. Rodent models of maternal obesity have shown that the offspring have increased systolic blood pressure, elevated triglyceride and cholesterol levels [41]. Similarly, offspring of normally fed dams cross-fostered and suckled by high fat-fed dams developed hypertension similar to those exposed to high fat during pregnancy [40]. These rats display endothelial dysfunction as well as abnormal aortic elasticity [83]. The consistently elevated blood pressure in offspring of obese dams was attributed to sympathetic over-activation, with altered heart rate variability and abnormal baroreceptor responsiveness [84]. As with insulin resistance, fat feeding in the absence of maternal obesity led to altered sympathetic control of cardiovascular function and hypertension [85].

### Endocrine Pancreas

In models of maternal under-nutrition reductions of beta cell mass and insulin content are associated with a reduction in insulin secretion. In contrast, offspring of dams fed a high fat diet through gestation alone showed altered neonatal islets with increased alpha cell number and size and with an opposite effect on the beta cell [86]. Offspring of obese dams fed an obesogenic diet throughout gestation and lactation were hyperinsulinemic at 3 months of age [41], which was associated with increased pancreatic insulin content, increased islet number and increased beta cell mass in early life [87], which declined with age due to persistent stimulation [63]. Similar observations were recorded in sheep where at lambing reduced offspring beta cell numbers was associated with an increase in beta cell apoptosis [88]. In a model of paternal high fat feeding impaired beta cell function in female offspring early in life was associated with reduced islet area and insulin secretion following a glucose challenge [89•].

## Molecular Mechanisms

A number of underlying mechanisms have been investigated in models of developmental programming. Markers of oxidative stress have been associated with obesity and diabetes and recent studies have suggested that reactive oxygen species production may be a key event preceding the onset of obesity in response to maternal nutrition in the placenta [90], fetal skeletal muscle [91] and fetal liver [53]. Interestingly, antioxidant supplementation to dams fed a western diet was able to reduce oxidative stress, inflammation, and adiposity in the embryos [92•]. The mitochondrion is particularly sensitive to early developmental programming and mutations in mitochondrial DNA persist through generations influencing long-term cellular functions including adaptive thermogenesis. Mitochondrial dysfunction has been reported as early as embryogenesis in obese mothers, with these embryos having increased mitochondrial membrane potential, higher levels of oxidative phosphorylation and increased reactive oxygen species production [93]. Disruptions in the electron transport chain have also been demonstrated in skeletal muscle and liver of offspring from over-nourished mothers [79, 94].

Epigenetic dysregulation has been reported to mediate the effects of early nutrition on adult disease susceptibility, of which DNA methylation is the most common mechanism studied. Methyl donors are sourced from the diet and methylation patterns are established during development. Therefore methyl donor imbalances could alter epigenetic patterns and increase an individual to metabolic disease in later life [95•]. In the pancreas of rat pups reared in small litters, an epigenetic alteration of the insulin 2 gene reduced its expression and was associated with reduced glucose stimulated insulin secretion [96]. Hypermethylation of the hypothalamic POMC promoter was observed in this model [97]. In aortic endothelial cells, hyperglycemia results in the activation of nuclear factor κB increasing its expression. Changes such as these are clearly important when considering that diabetes is a major independent risk factor for atherosclerotic cardiovascular disease [98]. Data is emerging on the transgenerational effects of maternal obesity and diet, with maternal diet influencing body length and insulin sensitivity in second and third generation mice [99]. The transmission solely through the paternal lineage suggests epigenetic programming of the sperm epigenome [89•]. Therefore these studies highlight how maternal nutrition can influence health of future generations and may explain the rapid increase in obesity prevalence through generations.

## Future Direction and Clinical Interventions

Evidence to date highlights the importance of the obese pre- and postnatal environment and the consequences of developmental programming on an infant’s susceptibility to metabolic disorders in later life. Clearly, the use of animal models has been extremely useful for dissecting the contributory factors and critical time windows. Over the next few years, it is likely that mechanisms will be identified by which early life programming determines the set point of energy balance and how the numerous brain circuits and peripheral endpoints are integrated and regulated so that energy expenditure and energy intake are matched. Improved nutritional awareness of the mother and father are essential. Dietary restriction and weight loss prior to pregnancy are proven strategies to improve infant health outcome, but therapeutic interventions will likely be of use in obese pregnant women. Understanding the programming signals related to maternal over-nutrition will allow the improved healthcare of both mother and infant.
